# Cocoa-flavanols enhance moderate-intensity pulmonary $\dot{V}{\text{O}}_{2}$ kinetics but not exercise tolerance in sedentary middle-aged adults

**DOI:** 10.1007/s00421-021-04682-9

**Published:** 2021-05-10

**Authors:** Daniel G. Sadler, Richard Draijer, Claire E. Stewart, Helen Jones, Simon Marwood, Dick H. J. Thijssen

**Affiliations:** 1 School of Sport and Exercise Science, Liverpool John Moores University, Byrom Street, Liverpool, L3 3AF UK; 2 School of Health Sciences, Liverpool Hope University, Liverpool, UK; 3 Unilever Research & Development, Olivier van Noortlaan 120, 3133 AT Vlaardingen, The Netherlands

**Keywords:** Flavanols, Oxygen uptake kinetics, Heart rate, Exercise tolerance, Middle-age

## Abstract

**Introduction:**

Cocoa flavanols (CF) may exert health benefits through their potent vasodilatory effects, which are perpetuated by elevations in nitric oxide (NO) bioavailability. These vasodilatory effects may contribute to improved delivery of blood and oxygen (O_2_) to exercising muscle.

**Purpose:**

Therefore, the objective of this study was to examine how CF supplementation impacts pulmonary O_2_ uptake ($\dot{V}{\text{O}}_{2}$) kinetics and exercise tolerance in sedentary middle-aged adults.

**Methods:**

We employed a double-blind cross-over, placebo-controlled design whereby 17 participants (11 male, 6 female; mean ± SD, 45 ± 6 years) randomly received either 7 days of daily CF (400 mg) or placebo (PL) supplementation. On day 7, participants completed a series of ‘step’ moderate- and severe-intensity exercise tests for the determination of $\dot{V}{\text{O}}_{2}$ kinetics.

**Results:**

During moderate-intensity exercise, the time constant of the phase II $\dot{V}{\text{O}}_{2}$ kinetics ($\tau \dot{V}{\text{O}}_{2}$) was decreased by 15% in CF as compared to PL (mean ± SD; PL 40 ± 12 s vs. CF 34 ± 9 s, *P* = 0.019), with no differences in the amplitude of $\dot{V}{\text{O}}_{2}$ (*A*$\dot{V}{\text{O}}_{2}$; PL 0.77 ± 0.32 l min^−1^ vs. CF 0.79 ± 0.34 l min^−1^, *P* = 0.263). However, during severe-intensity exercise, $\tau \dot{V}{\text{O}}_{2}$, the amplitude of the slow component (${\text{SC}}\dot{V}{\text{O}}_{2}$) and exercise tolerance (PL 435 ± 58 s vs. CF 424 ± 47 s, *P* = 0.480) were unchanged between conditions.

**Conclusion:**

Our data show that acute CF supplementation enhanced $\dot{V}{\text{O}}_{2}$ kinetics during moderate-, but not severe-intensity exercise in middle-aged participants. These novel effects of CFs, in this demographic, may contribute to improved tolerance of moderate-activity physical activities, which appear commonly present in daily life.

**Trial registration:**

Registered under ClinicalTrials.gov Identifier no. NCT04370353, 30/04/20 retrospectively registered

## Introduction

Skeletal muscle $\dot{V}{\text{O}}_{2}$ contraction and force production form the basis for the ability to perform physical activity, both for daily life activities as well as during sports-related events. Repeated muscle contractions require continuous regeneration of adenosine triphosphate (ATP). The production of ATP during (prolonged) physical activity is driven through the mechanism of oxidative phosphorylation, which depends on sufficient availability of oxygen (O_2_) amongst other key substrates (Poole et al. 2008). Impairment to pathways involved in the delivery of O_2_ to working skeletal muscle, like that observed in older and physically inactive adults, leads to slower rates of pulmonary O_2_ uptake ($\dot{V}{\text{O}}_{2}$) and therefore greater O_2_ deficit (Cunningham and Paterson 1994; DeLorey et al. 2004a; Dumanoir et al. 2010; Whipp and Rossiter 2013; George et al. 2018). Slower $\dot{V}{\text{O}}_{2}$ kinetics in response to physical activity are associated with lower exercise tolerance (Grassi et al. 2011; Goulding et al. 2017, 2018), and may affect the capacity to perform daily life activities that require moderate-intensity physical activity.

The slower dynamic adjustment of $\dot{V}{\text{O}}_{2}$ across a metabolic transient observed in older adults is thought to be due to a mismatch of O_2_ delivery to O_2_ utilisation (Murias et al. 2010; Murias and Paterson 2015; George et al. 2018). Indeed, attenuations in microvascular blood flow supply and distribution (and thus O_2_ delivery) within aged skeletal muscle are well documented (Muller-Delp et al. 2002; Musch et al. 2004; Behnke and Delp 2010; Dumanoir et al. 2010). These reductions in O_2_ delivery to active skeletal muscle are likely caused by impaired vascular endothelial function and diminished nitric oxide (NO) bioavailability (Muller-Delp et al. 2002; Woodman et al. 2002; Spier et al. 2004; Sindler et al. 2009). Interestingly, lifestyle interventions, such as exercise training and dietary strategies (Vanhatalo et al. 2010; Schreuder et al. 2015), have demonstrated potent effects to enhance NO bioavailability and improve endothelial function. Consequently, a number of studies have shown faster $\dot{V}{\text{O}}_{2}$ kinetics in concert with increased O_2_ availability (Murias et al. 2010; Bailey et al. 2015; Goulding et al. 2017).

Cocoa flavanols (CFs) represent a group of flavonoids present in cocoa derived from seeds of the fruit of the Theobroma cacao tree. Previous studies have found CFs (700–900 mg range) act primarily through the monomer (−)-epicatechin, to stimulate NO production, resulting in improved vasodilation and endothelial function in healthy adults (Schroeter et al. 2006; Davison et al. 2008; Berry et al. 2010). Given the direct impact of CFs on NO production and vascular endothelial function, and the negative effects of sedentary ageing on O_2_ delivery and $\dot{V}{\text{O}}_{2}$ kinetics at the onset of exercise, our objective was to test the hypothesis that, compared with placebo (PL), CF supplementation speeds phase II $\dot{V}{\text{O}}_{2}$ kinetics during moderate-intensity physical activity and increases exercise tolerance in healthy middle-aged individuals.

## Methodology

### Participants

**Fig. 1:**
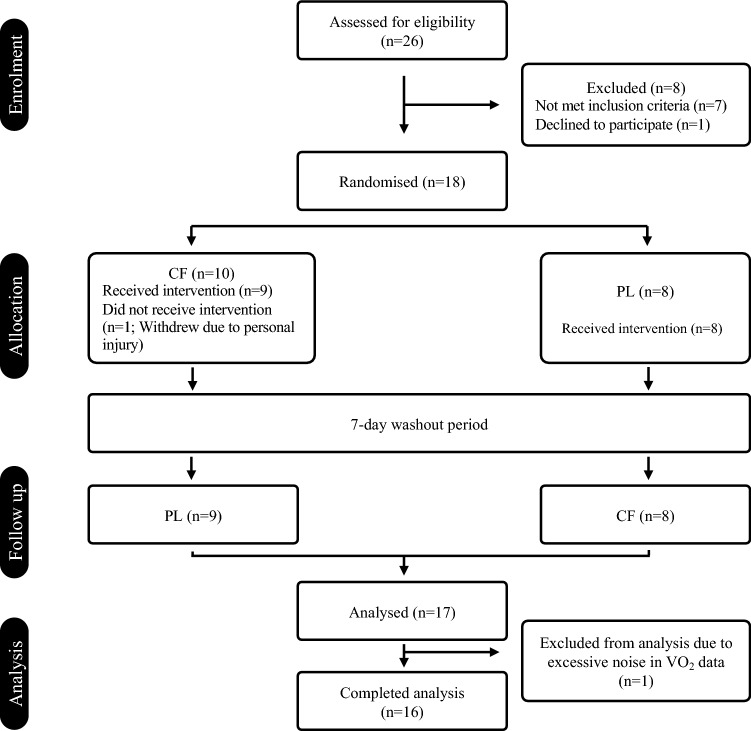
CONSORT diagram showing the flow of participants through each stage of the randomised trial

Seventeen healthy middle-aged adults (11 male: mean ± SD, age 45 ± 6 years; body mass 87.7 ± 13.5 kg; height 1.75 ± 0.07 m; and 6 female: aged 47 ± 5 years; body mass 68.2 ± 17.7 kg; height 1.62 ± 0.09 m) volunteered and gave written informed consent to participate in the study (see Fig. 1). All procedures conformed to the Declaration of Helsinki and were approved by Liverpool John Moores University Research Ethics Committee (approval reference number: 18/SPS/014). Participants engaged in less than two hours of structured exercise training per week. All participants were non-smokers and had no history of cardiovascular, respiratory or metabolic diseases. Participants were not taking any dietary supplements or medication.

Participants reported to the laboratory at least 3 h postprandial in a rested state, having completed no strenuous exercise within the previous 24 h and avoided alcohol and caffeine for 24 and 6 h, preceding each exercise test, respectively. Participants were advised to avoid consumption of flavonoid-rich foodstuffs (e.g. green tea, dark chocolate and berries) in the 24 h preceding each experimental trial.

### Procedures

Participants visited the temperature-controlled laboratory (19–22 °C) on four occasions during a 4–5-week period, with each test scheduled at the same time of day (± 1 h) and at least 48 h between visits. Participants completed two preliminary trials and two experimental trials. Exercise bouts were performed on an electrically operated cycle ergometer (Lode Corival, Groningen, The Netherlands). Saddle and handlebar height/angle were recorded at the first visit and replicated during each subsequent visit for each individual participant. Throughout all exercise tests, participants were instructed to maintain a cadence of 65–80 rev min^−1^, and exhaustion was defined as when the participants cadence dropped 10 rev min^−1^ below the target work rate. Time to exhaustion was measured to the nearest second (s) in all tests.

### Preliminary trial(s)

Upon arrival to the laboratory, participants’ height and weight were recorded. Subsequently, each participant undertook an incremental step test until the limit of tolerance to establish $\dot{V}{\text{O}}_{2}$ peak, the gas exchange threshold (GET) and the power outputs for later tests. The incremental step test consisted of 3-min of baseline pedalling at 0 W, followed by a continuous, stepped increase in power output of 30 or 25 W every minute (for males and females, respectively) until the limit of tolerance was established. Gas exchange and ventilatory variables were measured continuously at the mouth breath-by-breath throughout each test. $\dot{V}{\text{O}}_{2}$ peak was defined as the highest $\dot{V}{\text{O}}_{2}$ value obtained over 30 s. The GET was determined using a collection of previously established criteria (Beaver et al. 2016) including (1) a disproportionate rise in CO_2_ production ($\dot{V}{\text{CO}}_{2}$) relative to $\dot{V}{\text{O}}_{2}$; (2) an increase in minute ventilation ($\dot{V}{\text{E}}$) relative to $\dot{V}{\text{O}}_{2}$ (${{\dot{V}{\text{E}}} \mathord{\left/ {\vphantom {{\dot{V}{\text{E}}} {\dot{V}{\text{O}}_{2} }}} \right. \kern-\nulldelimiterspace} {\dot{V}{\text{O}}_{2} }}$) without an increase in $\dot{V}{\text{E}}$ relative to $\dot{V}{\text{CO}}_{2}$ (${{\dot{V}{\text{E}}} \mathord{\left/ {\vphantom {{\dot{V}{\text{E}}} {\dot{V}{\text{CO}}_{2} }}} \right. \kern-\nulldelimiterspace} {\dot{V}{\text{CO}}_{2} }}$); and (3) an increase in end tidal O_2_ tension without decreasing end tidal CO_2_ tension.

During the familiarisation trial (visit 2), participants were requested to perform two bouts of severe-intensity exercise at a fixed power output to exhaustion (e.g. T_lim_), each separated by 45 min of seated rest. The power outputs of these severe-intensity bouts were selected based upon performance during the incremental test and were calculated to be 60% ∆ (i.e., a work rate calculated to require 60% of the difference between GET and $\dot{V}{\text{O}}_{2}$ peak). On occasion, adjustments were made to the power output of the subsequent exercise tests based upon performance in the familiarisation trials; the prescribed power output was lowered for participants who failed to exercise for up to 360 s during the severe-intensity bouts.

**Fig. 2:**
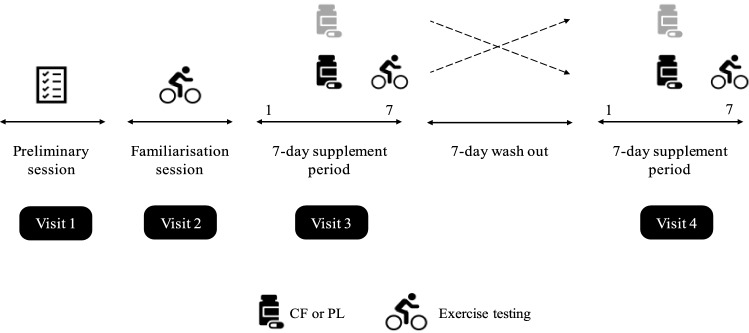
Schematic of experimental design

After completion of the familiarisation trial, participants were randomly assigned (computer generated), using a double-blind cross-over design (Fig. 2), to receive seven consecutive days of CF supplementation or a PL that was matched for caffeine and theobromine content. Nine participants began with the CF condition, and eight participants began with the PL condition. Participants were advised to consume four capsules daily. Each CF capsule contained 316 mg CocoActiv (Naturex, Netherlands; ~ 100 mg total flavanols of which 22 mg DP1 = catechin + epicatechin) whereas PL capsules contained 0 mg CocoActiv product. This CF dose was selected based on the knowledge that ~ 400 mg CF’s are required to improve vascular function during exercise (Decroix et al. 2018a). Both PL and CF capsules contained 2.9 mg caffeine and 22.5 mg theobromine (Fagron, Netherlands). Remaining empty volumes of PL and CF capsules were filled with microcrystalline cellulose (Fagron, Netherlands). Two capsules were taken in the morning and two in the evening following ingestion of a mixed meal (Cifuentes-Gomez et al. 2015). A 7-day wash-out period separated the supplementation periods and the order between CF and PL supplementation was randomised. Throughout the study period participants were instructed to maintain their normal daily activities and diet. Participants kept a food dairy and were instructed to consume an identical diet in the two periods of exercise testing. Physical activity levels were measured by accelerometery in the 6 days preceding testing via a hip-mounted activity monitor (Actigraph GT3X).

### Experimental trials

On the 7th day of supplementation, participants were advised to consume four capsules 45 min prior to arrival at the laboratory. The supplementation protocol was chosen so that participants commenced exercise testing ~ 90 min following supplement ingestion, which coincided with reported peak plasma flavanol concentrations (Cifuentes-Gomez et al. 2015). The participants completed a series of separate “step” exercise tests from an unloaded (0 W) baseline to moderate or severe-intensity work rates for the determination of pulmonary $\dot{V}{\text{O}}_{2}$ kinetics. Tests began with 3 min of 0 W baseline cycling, before a step change in power output to 80% GET for 6 min or 60% ∆ until T_lim_. Participants sequentially completed three bouts of moderate- and one bout of severe-intensity exercise, each separated by 10 min of passive recovery. This protocol was employed with the knowledge that multiple bouts of moderate-intensity exercise do not impact the $\dot{V}{\text{O}}_{2}$ kinetics of subsequent moderate- and heavy-intensity exercise (Burnley et al. 2000; Spencer et al. 2011).

### Measurements

After arrival to the laboratory, participants underwent an assessment of the previous 7 days physical activity levels and sedentary behaviour by the International Physical Activity Questionnaire (IPAQ) and by accelerometery (ActiGraph GTX3) (data not shown). Following 10 min of seated rest, participants blood pressure was measured in the brachial artery. Blood pressure was measured three times and the mean of the responses was recorded.

During all exercise tests, pulmonary gas exchange and ventilation were measured at the mouth breath-by-breath using a metabolic cart (Jaeger Oxycon Pro, Hoechberg, Germany). Participants wore a facemask and breathed through a low dead space (90 ml), low resistance (0.75 mmHg l^−1^ s^−1^ at 15 l/s) impeller turbine assembly (Jaeger Triple V, Hoechberg, Germany). The inspired and expired gas volumes and gas concentration signals were continuously sampled at 100 Hz, the latter using paramagnetic (O_2_) and infrared (CO_2_) analysers (Jaeger Oxycon Pro, Hoechberg, Germany) via a capillary line connected to the mouthpiece. These analysers were calibrated before each test with gases of known concentrations (16% O_2_ and 4% CO_2_), and the turbine volume transducer was calibrated using a 3-L syringe (Hans Rudolph, Kansas City, MO). The volume and concentration signals were time aligned by accounting for the delay in capillary gas transit and analyser rise time relative to the volume signal. Breath-by-breath fluctuations in lung gas stores were corrected for by computer algorithms (Beaver et al. 1981). Heart rate was measured during all tests via short-range radiotelemetry (Polar H10, Polar Electro, Kempele, Finland). During one of the transitions to moderate- and severe-intensity exercise for both supplementation periods, a blood sample was collected from a fingertip over the last 30 s preceding the step transition in work rate and within the last 15 s of exercise. Blood samples were immediately analysed using a hand-held device (Lactate Pro, Nova Biomedical, USA) to determine blood lactate concentration. Blood lactate accumulation was calculated as the difference between blood lactate at end exercise and blood lactate at baseline.

### Data analysis

Breath-by-breath $\dot{V}{\text{O}}_{2}$ data were edited to remove data points lying more than three standard deviations (SD) outside the local 5-breath mean (Lamarra et al. 1987). The resultant data were then linearly interpolated to provide second-by-second values. For $\dot{V}{\text{O}}_{2}$ and heart rate data in response to moderate exercise transitions, second-by-second data for the three transitions were averaged together to produce a single dataset. The severe-intensity exercise bout for each condition was not repeated and was modelled separately. For each exercise transition, the first 20 s of data after the onset of exercise (i.e., the cardiodynamic or phase I response) was deleted (McNarry et al. 2012; Benson et al. 2017) and a mono-exponential model (Eq. 1) with time delay was then fitted to the data (Whipp and Rossiter 2013), as follows:

1 $$\dot{V}{\text{O}}_{2} = \dot{V}{\text{O}}_{{2({\text{b}})}} + A\dot{V}{\text{O}}_{2} \left( {1 - e^{{ - (t - {\text{TD}}\dot{V}{\text{O}}_{2} )}} } \right)$$

where $\dot{V}{\text{O}}_{2}$(*t*) is the $\dot{V}{\text{O}}_{2}$ at any time *t*, $\dot{V}{\text{O}}_{{2{\text{b}}}}$ is the baseline $\dot{V}{\text{O}}_{2}$, which was taken as the mean $\dot{V}{\text{O}}_{2}$ over the final 30 s of the baseline period preceding the transition, $A\dot{V}{\text{O}}_{2}$is the amplitude of the primary response above baseline, ${\text{TD}}_{{\dot{V}{\text{O}}_{2} }}$ is the time delay of the primary response relative to the onset of exercise, and $\tau \dot{V}{\text{O}}_{2}$ is the time constant of the primary response. For moderate-intensity exercise, data were modelled to 360 s. For severe-intensity exercise, the onset of the $\dot{V}{\text{O}}_{2}$slow component (${\text{TD}}_{{{\text{SC}}\dot{V}{\text{O}}_{2} }}$) was determined using purpose-designed programming in Microsoft Excel (Microsoft Corporation, Redmond, WA, USA), which iteratively fits a monoexponential function to the $\dot{V}{\text{O}}_{2}$ data, starting at 60 s until the window encompasses the entire response. The resulting primary time constants are plotted against time, and the ${\text{TD}}_{{{\text{SC}}\dot{V}{\text{O}}_{2} }}$ was identified as the point at which $\tau \dot{V}{\text{O}}_{2}$ consistently deviates from a previously ‘flat’ profile, and the demonstration of a local threshold in the *χ*^2^ value (Rossiter et al. 2001). This method allows the fitting of Eq. (1) to the primary component of the response isolated from the slow component, thus avoiding the possibility of arbitrarily parameterizing the slow component. The amplitude of the $\dot{V}{\text{O}}_{2}$ slow component was determined by calculating the difference between the end-exercise $\dot{V}{\text{O}}_{2}$ (i.e. mean $\dot{V}{\text{O}}_{2}$ over final 30 s of exercise) and ($A\dot{V}{\text{O}}_{2} + \dot{V}{\text{O}}_{{2{\text{b}}}}$). In instances where exercise duration was too short to allow the slow component to be discerned the $\dot{V}{\text{O}}_{2}$ response was modelled using Eq. (1) to the end of exercise and the slow component was assigned a value of 0.

Heart rate kinetics were modelled for each exercise transition using a monoexponential function (Eq. 2) with the response constrained to the start of exercise (at *t* = 0; i.e., with no time delay):

2 $${\text{HR}}_{{({\text{t}})}} = {\text{HR}}_{{\text{b}}} + A{\text{HR}} \times \left( {1 - e^{{({t \mathord{\left/ {\vphantom {t {\tau {\text{HR}}}}} \right. \kern-\nulldelimiterspace} {\tau {\text{HR}}}})}} } \right)$$

where HR_b_ is the mean HR measured over the final 30 s of baseline cycling, and *A*HR and *τ*HR are the amplitude and the time constant of the response, respectively.

### Statistics

Based on previous knowledge of a meaningful change in $\tau \dot{V}{\text{O}}_{2}$ during intervention studies (5 s), and a common standard deviation of 4.3 s (Benson et al. 2017), the necessary calculated sample size was 12. Differences in the cardiorespiratory variables between conditions were determined with two-tailed, paired-samples *t* tests (GraphPad, Prism, USA). Data are presented as means ± SD. Statistical significance was accepted when *P* < 0.05.

## Results

Peak $\dot{V}{\text{O}}_{2}$ was 2.45 ± 0.61 l min^−1^ (28.1 ± 5.7 ml kg^−1^ min^−1^), with the mean GET occurring at 1.51 ± 0.46 l min^−1^ (108 ± 39 W). The peak work rate attained from the incremental test was 207 ± 49 W and the work rates calculated to require 80% of the GET and 60% ∆ were 87 ± 29 W and 166 ± 40 W, respectively.

### Heart rate kinetics, blood lactate profiles and blood pressure

There were no differences in the primary *τ*HR between PL and CF for moderate- or severe-intensity bouts (*P* = 0.219 and 0.956, respectively, Table 1). Despite significant changes in blood lactate concentrations at T_lim_ compared to baseline (*P* < 0.05; Table 1), there were no significant differences in blood (lactate) from pre- to post-exercise between conditions during moderate- and severe-intensity exercise (see Table 1). Overall, there were no differences between resting systolic (PL 128 ± 12 mmHg vs. CF 127 ± 12 mmHg, *P* = 0.66) or diastolic (PL 78 ± 7 mmHg vs. 78 ± 7 mmHg, *P* = 0.75) blood pressure following either PL or CF administration.

**Table 1:** Heart rate and blood lactate responses during moderate- and severe-intensity exercise following CF and PL supplementation

Parameter	HR_b_ (b min ^−1^)	*A*HR (b min^−1^)	*τ*HR (s)	End exercise HR (b min^−1^)	Baseline blood lactate (mM)	End exercise blood lactate (mM)	∆ blood lactate (mM)	Blood lactate at exhaustion (mM)
Moderate-intensity exercise								
PL	83 ± 13	31 ± 8	53 ± 22	114 ± 16	1.5 ± 0.7	2.6 ± 0.5	1.2 ± 0.9	–
CF	83 ± 14	32 ± 8	47 ± 13	115 ± 18	1.3 ± 0.4	2.5 ± 0.7	1.3 ± 0.8	–
Severe-intensity exercise								
PL	89 ± 15	69 ± 16	89 ± 17	159 ± 14	1.9 ± 0.9	8.8 ± 2.0	7.4 ± 2.5	9.5 ± 2.3^#^
CF	92 ± 17	67 ± 17	89 ± 29	160 ± 17	1.8 ± 0.9	8.4 ± 2.3	7.1 ± 2.8	9.7 ± 1.9^#^

Values are mean ± SD

*HR*_*b*,_ baseline heart rate, *AHR* amplitude of the fundamental response, *τHR* time constant of the fundamental response, *PL* placebo, *CF* cocoa flavanol

^#^Significantly different from baseline blood lactate (*P* < 0.05)

### $\dot{V}{\text{O}}_{2}$ kinetics and exercise tolerance

**Fig. 3:**
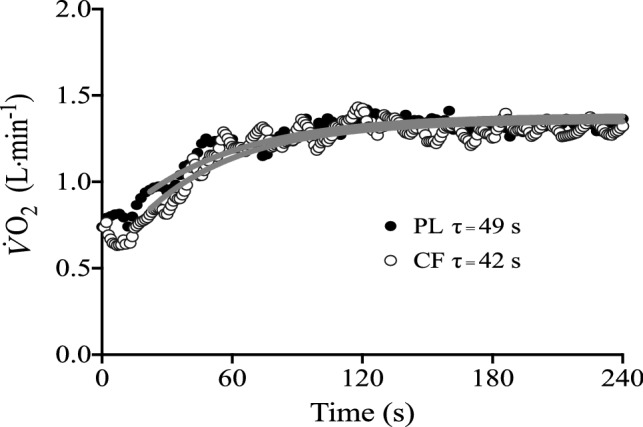
Pulmonary $\dot{V}{\text{O}}_{2}$ and best-fit modelled responses of a representative participant to moderate-intensity exercise following PL (solid black circles) and CF (clear circles) supplementation. $\tau \dot{V}{\text{O}}_{2}$ values are displayed for each transition, with the solid grey lines representing the modelled fits

The $\dot{V}{\text{O}}_{2}$ kinetic parameters for moderate-intensity exercise are presented in Table 2, and the $\dot{V}{\text{O}}_{2}$ response of a representative participant to moderate-intensity exercise is shown in Fig. 3. Compared with PL, $\tau \dot{V}{\text{O}}_{2}$ was smaller during moderate-intensity exercise following CF supplementation (PL 40 ± 12 s vs. CF 34 ± 9 s, *P* = 0.019). However, there were no differences in $\dot{V}{\text{O}}_{{2{\text{b}}}}$ (*P* = 0.175), $A\dot{V}{\text{O}}_{2}$ (*P* = 0.263), ${\text{TD}}\dot{V}{\text{O}}_{2}$ (*P* = 0.961) or end exercise $\dot{V}{\text{O}}_{2}$ (*P* = 0.565) between PL and CF.

**Table 2:** Pulmonary O_2_ uptake responses to moderate- and severe-intensity exercise following CF and PL supplementation

Parameter	$\dot{V}{\text{O}}_{{2{\text{b}}}}$ (l min^−1^)	$A\dot{V}{\text{O}}_{2}$ (l min^−1^)	${\text{TD}}\dot{V}{\text{O}}_{2}$ (s)	$\tau \dot{V}{\text{O}}_{2}$ (s)	End exercise $\dot{V}{\text{O}}_{2}$ (l min^−1^)	${\text{TD}}_{{{\text{SC}}\dot{V}{\text{O}}_{2} }}$(s)	${\text{SC}}\dot{V}{\text{O}}_{2}$ (l min^−1^)	T_lim_ (s)
Moderate-intensity exercise								
PL	0.69 ± 0.12	0.77 ± 0.32	13 ± 6	40 ± 12	1.50 ± 0.35	–	–	–
CF	0.66 ± 0.13	0.79 ± 0.34	13 ± 7	34 ± 9*	1.50 ± 0.38	–	–	–
Severe-intensity exercise								
PL	0.78 ± 0.14	1.40 ± 0. 40	17 ± 4	27 ± 9	2.60 ± 0.66	110 ± 15	0.50 ± 0.20	435 ± 58
CF	0.74 ± 0.13	1.50 ± 0.52	16 ± 4	28 ± 6	2.60 ± 0.65	95 ± 13*	0.50 ± 0.20	424 ± 47

Values are mean ± SD

$\dot{V}O_{2b}$ baseline oxygen uptake, $A\dot{V}O_{2}$ amplitude of the fundamental response, $TD\dot{V}O_{2}$ time delay of the fundamental response, $\tau \dot{V}O_{2}$ time constant of the fundamental response, $TD_{{SC\dot{V}O_{2} }}$ time delay of the $\dot{V}{\text{O}}_{2}$ slow component, $SC\dot{V}O_{2}$ magnitude of the slow component, *T*_*lim*_ limit of exercise tolerance, *PL* placebo, *CF* cocoa flavanol

*Significantly different from PL (*P* < 0.05)

**Fig. 4:**
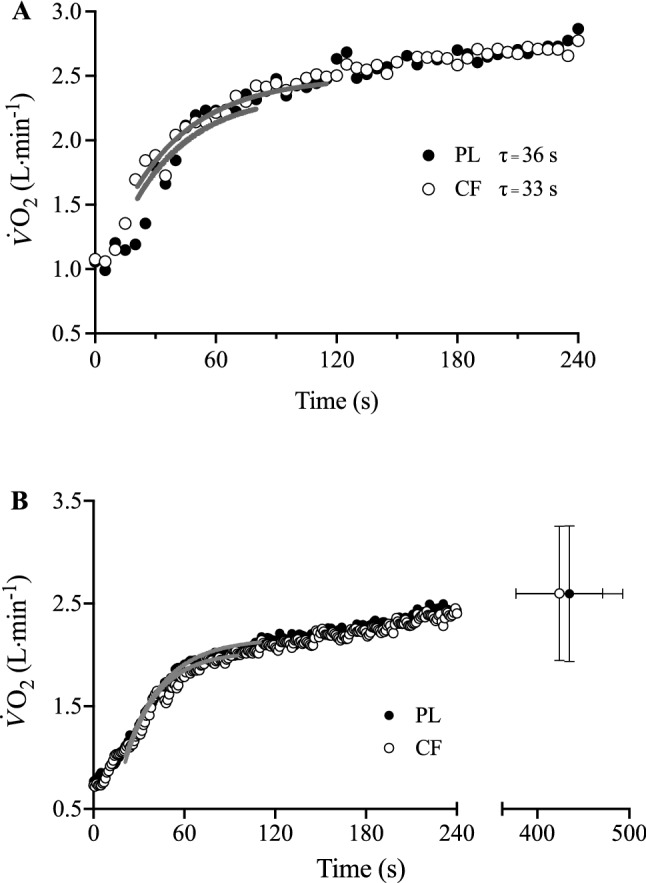
Pulmonary $\dot{V}{\text{O}}_{2}$ and best-fit modelled responses to severe-intensity exercise following PL (solid black circles) and CF (clear black circles) supplementation. Panel **a** Pulmonary $\dot{V}{\text{O}}_{2}$ responses of a representative participant displayed with associated $\tau \dot{V}{\text{O}}_{2}$. Panel **b** Group mean $\dot{V}{\text{O}}_{2}$ responses during the rest-to-exercise transition following PL and CF supplementation. Group mean ± SD $\dot{V}{\text{O}}_{2}$ at limit of exercise tolerance also shown. Solid grey lines represent the modelled fits

The pulmonary $\dot{V}{\text{O}}_{2}$ response to severe-intensity exercise for a representative participant is shown in Fig. 4a and group mean responses are shown in Fig. 4b. The associated modelled parameters are presented in Table 2. No impact of CF supplementation on the $\tau \dot{V}{\text{O}}_{2}$ (*P* = 0.799) for exercise initiated at 60% ∆ over PL was evident. There were no differences in $\dot{V}{\text{O}}_{{2{\text{b}}}}$ (*P* = 0.246), $A\dot{V}{\text{O}}_{2}$ (*P* = 0.427), ${\text{TD}}\dot{V}{\text{O}}_{2}$ (*P* = 0.617), ${\text{SC}}\dot{V}{\text{O}}_{2}$ (*P* = 0.887) or end exercise $\dot{V}{\text{O}}_{2}$ (*P* = 0.954) between conditions. ${\text{TD}}_{{{\text{SC}}\dot{V}{\text{O}}_{2} }}$ was lower following CF vs. PL supplementation (PL 110 ± 15 s vs. CF 95 ± 13 s, *P* = 0.002). Both end-exercise $\dot{V}{\text{O}}_{2}$ (*P* = 0.959) and T_lim_ (*P* = 0.480) were not significantly different following PL and CF supplementation during severe-intensity exercise (see Table 2).

## Discussion

The purpose of this study was to examine the impact of CFs on pulmonary $\dot{V}{\text{O}}_{2}$ kinetics during two intensities of cycling exercise in healthy, normotensive middle-aged individuals. Congruent with our hypothesis, the major finding of this study was that 7-days CF supplementation sped pulmonary $\dot{V}{\text{O}}_{2}$ kinetics during moderate-intensity exercise as demonstrated by a significant reduction in $\tau \dot{V}{\text{O}}_{2}$. These effects of CFs, however, were not apparent during severe-intensity exercise when compared with a PL. Ultimately, the findings of the present study may have clinical potential in contributing to improved tolerance of daily life activity in middle-aged adults.

### Effects of CFs on the physiological responses to moderate-intensity exercise

This study is the first to investigate whether CFs modulate pulmonary $\dot{V}{\text{O}}_{2}$ kinetics. We show that 7 days CF supplementation significantly reduced the $\tau \dot{V}{\text{O}}_{2}$ (40 s vs. 34 s) associated with the transition from unloaded to moderate-intensity cycling in middle-aged adults. Notably, the magnitude of change in $\tau \dot{V}{\text{O}}_{2}$ (~ 6 s) reported is important, as it exceeds the minimum physiologically relevant change of ~ 5 s (Benson et al. 2017). The reduction in $\tau \dot{V}{\text{O}}_{2}$ observed after CF supplementation in our middle-aged individuals reflects a shift towards values typically observed in younger healthy individuals (Grassi et al. 2009), whereby $\dot{V}{\text{O}}_{2}$ kinetics are not limited by O_2_ delivery per se (Poole and Jones 2012). Theoretically, a lowered $\tau \dot{V}{\text{O}}_{2}$ would reduce the O_2_ deficit incurred during the exercise transition, thereby causing less perturbations to the intracellular milieu (i.e., ∆ phosphocreatine, ADP, H^+^, inorganic phosphate, glycogen) and enhancing exercise tolerance (Grassi et al. 2011; Goulding et al. 2017, 2018). Therefore, our data suggest CFs may lower the O_2_ deficit incurred during moderate-intensity activity by negating age-associated impairments to pulmonary $\dot{V}{\text{O}}_{2}$ kinetics.

Since the purpose of the study was to examine the impact of CFs on $\dot{V}{\text{O}}_{2}$ kinetics, our data raise the question about the potential underlying mechanisms contributing to the lowered $\tau \dot{V}{\text{O}}_{2}$ with CF supplementation. It is acknowledged $\tau \dot{V}{\text{O}}_{2}$ is sensitive to manipulations in O_2_ delivery (DeLorey et al. 2004b; Gurd et al. 2009), and, that the slowing of $\dot{V}{\text{O}}_{2}$ kinetics with advancing age occurs at least partly as a consequence of lowered O_2_ availability in oxidative skeletal muscle (DeLorey et al. 2004a; Musch et al. 2004; Behnke and Delp 2010). Given that CFs exert potent NO-dependent vasodilatory effects (Schroeter et al. 2006; Cifuentes-Gomez et al. 2015; Decroix et al. 2018a), CF supplementation may have sped $\dot{V}{\text{O}}_{2}$ kinetics by augmenting muscle blood flow and O_2_ availability. Although, it is important to acknowledge CFs can alter indices of mitochondrial biogenesis and function (Taub et al. 2012; Kopustinskiene et al. 2015), as well as lower markers of oxidative stress (Ahmed et al. 2020). Together these factors may also influence $\dot{V}{\text{O}}_{2}$ responses to exercise by augmenting the capacity for O_2_ utilsisation and delivery. Clearly, futher work is required to determine the mechanisms by which CFs may regulate blood flow and changes in $\dot{V}{\text{O}}_{2}$ kinetics.

In spite of differences in the kinetics of $\dot{V}{\text{O}}_{2}$, no changes in the O_2_ cost of moderate-intensity exercise were observed after CF supplementation. Similarly, Patel et al. (2015) demonstrated no significant reduction in $\dot{V}{\text{O}}_{2}$ during twenty minutes of moderate-intensity cycling after 14 days dark chocolate supplementation (Patel et al. 2015). Together these findings contrast those published employing alternate dietary means of augmenting NO bioavailability, such as dietary nitrate, which reduces the O_2_ cost of moderate-intensity activity (Larsen et al. 2007; Bailey et al. 2009; Vanhatalo et al. 2010; Lansley et al. 2011). Such discrepancies may be explained by recent evidence linking dietary nitrate to improved contractile function (Bailey et al. 2019), an effect that has not been reported with CF supplementation. Possibly, the mechanisms by which CFs impact physiological responses to exercise relate to muscle O_2_ delivery rather than contractile function. Given that we did not measure NO or redox biomarkers, it is not clear to what extent CFs sped phase II $\dot{V}{\text{O}}_{2}$ kinetics through processes associated with reactive O_2_ and nitrogen species. Additional research will help delineate CFs mode of action in the context of exercise.

### Effects of CFs on the physiological responses to severe-intensity exercise

In contrast to our observations during moderate-intensity exercise, acute CF supplementation had no measurable impact on pulmonary $\dot{V}{\text{O}}_{2}$ kinetics during severe-intensity cycling. For instance, the $\tau \dot{V}{\text{O}}_{2}$ of the phase II response was similar between PL and CF (27 s vs. 28 s, respectively). The kinetics of $\dot{V}{\text{O}}_{2}$ are considered to be an important determinant of exercise tolerance (Whipp and Ward 1992; Grassi et al. 2011). In line with this principle, we observed no effect of CF supplementation on T_lim_ during severe-intensity exercise. Whilst no previous studies have examined the impact of CF supplementation on $\dot{V}{\text{O}}_{2}$ kinetics in the severe-intensity exercise domain, a number have studied their effects on exercise performance. Our findings corroborate these data showing no beneficial impact of acute or sub-chronic CF supplementation on time-trial or time-to-exhaustion performance in healthy male adults (Allgrove et al. 2011; Davison et al. 2012; Peschek et al. 2013; Stellingwerff et al. 2014; Decroix et al. 2018b).

Our data demonstrate divergent effects of CFs on $\dot{V}{\text{O}}_{2}$ kinetics between moderate- and severe-intensity exercise domains. Given that the pattern of muscle-fibre activation within moderate- and severe-intensity exercise domains differs (type I and type II predominant, respectively) (Krustrup et al. 2004), future studies should investigate a potential muscle fibre-type dependency of CF supplementation on the physiological responses to exercise. Another potential explanation for the differences between exercise intensity domains presented herein relates to the dose of CFs administered. Recent published evidence suggests that the 400 mg CF prescribed is the minimum dose necessary to exert beneficial effects during exercise (Decroix et al. 2018a). Therefore, the dose used in the present study may not have been high enough to raise blood flow sufficiently to detect a measurable effect upon $\dot{V}{\text{O}}_{2}$ kinetics during severe-intensity exercise. In addition, CFs had no beneficial impact on resting systolic or diastolic blood pressure over PL, which may be attributable to insufficient dosage and the normotensive population studied (Hooper et al. 2012). Besides, another limitation of the study is that only a single bout of severe-intensity exercise was conducted. As we were unable to feasibly include additional visits for testing, we could not carry out multiple severe-intensity bouts to enhance the signal-to-noise ratio of these $\dot{V}{\text{O}}_{2}$ responses and potentially detect differences between conditions. Finally, it is possible that prior exercise, through its effects on (muscle) perfusion, sped the phase II $\dot{V}{\text{O}}_{2}$ kinetic response during severe-intensity exercise in the population studied (Scheuermann et al. 2002).

## Conclusion

In the present study, seven days supplementation with a flavanol-rich cocoa-extract resulted in a reduced $\tau \dot{V}{\text{O}}_{2}$ during moderate-, but not severe-intensity exercise in normotensive, middle-aged adults. Whilst the O_2_ cost of exercise was similar between CF and PL conditions, the phase II $\dot{V}{\text{O}}_{2}$ kinetics were sped at the onset of moderate-intensity exercise after acute CF intake. Such effects on phase II $\dot{V}{\text{O}}_{2}$ kinetics were not found during severe-intensity exercise with CF. Therefore, CF supplementation may reduce the metabolic perturbations associated with moderate-intensity exercise in middle-aged adults through speeding phase II $\dot{V}{\text{O}}_{2}$ kinetics.

## Data Availability

The datasets generated during and/or analysed during the current study are available from the corresponding author on reasonable request.

## Abbreviations

*A*HR: Amplitude of the fundamental heart rate response
$A\dot{V}{\text{O}}_{2}$: Amplitude of the phase II oxygen uptake response
CF: Cocoa flavanol
GET: Gas exchange threshold
HR: Heart rate
HR_b_: Baseline heart rate
*τ*HR: Time constant of the fundamental heart rate response
NO: Nitric oxide
O_2_: Oxygen
PL: Placebo
${\text{SC}}\dot{V}{\text{O}}$: Magnitude of the slow component
$\tau \dot{V}{\text{O}}_{2}$: Time constant of the phase II response
${\text{TD}}_{{{\text{SC}}\dot{V}{\text{O}}_{2} }}$: Time delay of the $\dot{V}{\text{O}}_{2}$ slow component
${\text{TD}}\dot{V}{\text{O}}_{2}$: Time delay of the phase II response
T_lim_: Limit of exercise tolerance
$$\dot{V}{\text{O}}_{2}$$: Oxygen uptake
$\dot{V}{\text{O}}_{{2{\text{b}}}}$: Baseline oxygen uptake
